# Fast γ Photon Imaging for Inner Surface Defects Detecting

**DOI:** 10.3390/s21238134

**Published:** 2021-12-05

**Authors:** Min Yao, Guangdong Luo, Min Zhao, Ruipeng Guo, Jian Liu

**Affiliations:** 1 College of Automation Engineering, Nanjing University of Aeronautics and Astronautics, Nanjing 210016, China; guangdong@nuaa.edu.cn (G.L.); zhaomin2@nuaa.edu.cn (M.Z.); rpguo@nuaa.edu.cn (R.G.); jliu@nuaa.edu.cn (J.L.); 2 Nondestructive Detection and Monitoring Technology for High Speed Transportation Facilities, Key Laboratory of Ministry of Industry and Information Technolog, Nanjing 210016, China

**Keywords:** positron emission computed tomography, field-programmable gate array, iteration algorithm, internal defect detection, image reconstruction

## Abstract

Only a few effective methods can detect internal defects and monitor the internal state of complex structural parts. On the basis of the principle of PET (positron emission computed tomography), a new measurement method, using γ photon to detect defects of an inner surface, is proposed. This method has the characteristics of strong penetration, anti-corrosion and anti-interference. With the aim of improving detection accuracy and imaging speed, this study also proposes image reconstruction algorithms, combining the classic FBP (filtered back projection) with MLEM (maximum likelihood expectation Maximization) algorithm. The proposed scheme can reduce the number of iterations required, when imaging, to achieve the same image quality. According to the operational demands of FPGAs (field-programmable gate array), a BPML (back projection maximum likelihood) algorithm is adapted to the structural characteristics of an FPGA, which makes it feasible to test the proposed algorithms therein. Furthermore, edge detection and defect recognition are conducted after reconstructing the inner image. The effectiveness and superiority of the algorithm are verified, and the performance of the FPGA is evaluated by the experiments.

## 1. Introduction

Various types of industrial equipment operate in complex and severe environments, and they are usually subjected to fluctuant loads, corrosion and oxidation. Such conditions may lead to microdefects on their surfaces and even inside their bodies. For example, in complex devices, such as aircraft engines and engine blades, the defects always appear not only on the outer surface and subsurface but also on the surface of the inside cavities. Therefore, non-destructive methods should be able to detect and analyze the inner defects of an industrial device.

However, only a few of non-destructive methods can detect inner defects. Penetrant testing and electron microscopic testing can detect defects at the nanometer level in either metallic or nonmetallic materials [1]. However, these techniques can neither detect inner defects nor fully identify the exact location and depth of defects. Magnetic particle testing, acoustic emission testing and microwave testing techniques have fast detection speeds and high sensitivities, but they are vulnerable to magnetic field and noise interference [2]. The eddy current testing technique is highly precise in detecting defects on and near the surfaces of conductive materials, but only shallow surfaces can be penetrated [3]. Industrial CT (computed tomography) techniques use high-energy X-rays to penetrate a device during detection. However, the penetration capability of X-rays through metals is limited. X-ray energy must be increased to improve their penetration, but this is harmful to people’s health [4,5].

γ photons, produced by positron annihilation, have strong anti-interference properties, and they can work in extremely harsh environments, such as high-temperature and high-pressure conditions, at high speed [6,7]. These γ photons have strong penetrating ability and can even easily penetrate metallic materials [8]. In 2016, Xiao et al. (2016) studied the application of γ photon imaging to detect and capture the images of the inner cavities of structural components, and they investigated γ photon imaging scattering compensation in metallic materials [9]. Jiang et al. (2020) studied the 3D imaging of γ photons inside a dense metallic cavity [10]. Langford et al. (2016) designed a PET system to detect the fluid movement in a tube and mean flow velocity through it was measured [11]. Although uncertainty exists to the statistical nature of PEPT (positron emission particle tracking) method, multi-PEPT has been shown to be capable as a means of examining characteristics of a complex flow regime. Chen et al. (2020) designed a not-full-ring PET to measure the liquid distribution in a hydraulic cavity [12]. They verified that the not-full-ring scheme is feasible for imaging industrial parts.

**Figure 1:**
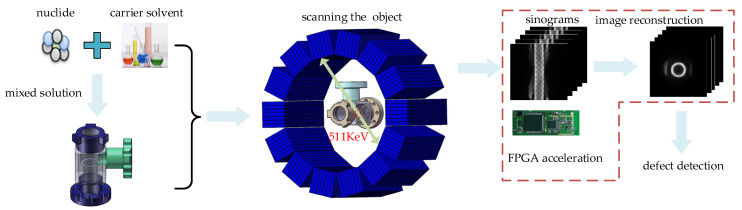
Diagram of the detection process.

Related studies have shown that γ photon imaging has the unique advantage of being capable of imaging inner defects [8,9,10,11,12]. Such detection process is shown in Figure 1. Nuclides with a positron liquid are injected into the cavity of an object. After positron annihilation, γ photons are generated. The detection rings can detect γ photons with energies of 511 KeV. Thus, γ photon pairs, within a certain energy window and certain time window, are detected as LOR (lines of response). The positions of positron annihilation and the nuclide concentration of positrons are estimated by LORs, which reflect the inner information. Then, inner defect imaging is achieved by image reconstruction. When defects exist on the surface of a cavity, they can be directly identified in the reconstructed images. 

It is obvious from the processing of image reconstruction that the speed of γ photon image reconstruction is very slow (about 10 min for a 156 × 156 × 52 3D image); so remedy this, parallel processing by FPGA is used in this study to accelerate γ photon imaging. This study proposes to combine the classic FBP (filtered-back projection) with MLEM (maximum likelihood expectation maximization) to build a BPML (back-projection maximum likelihood) image reconstruction algorithm. The proposed scheme can reduce the number of iterations without compromising image quality.

## 2. Tomographic Images Reconstructed in FPGA

**Figure 2:**
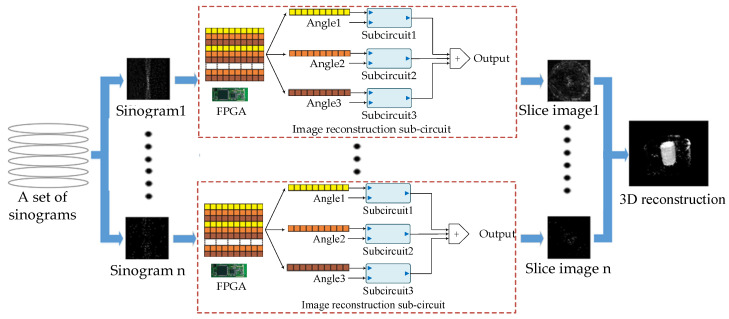
Parallel processing for image slice reconstruction.

After the labelled industrial object is scanned by the gamma photon detector, the LORs related to the inner radionuclide concentration distributions are obtained and stored in sinograms. An LOR is a pair of two 180-degree γ photons that meet the 511 KeV energy window and a certain time window [13,14]. Billions of LORs (depending on scanning time) saved in a group of parallel sinograms are recombined and reconstructed into tomographic images. As each single slice of the image reconstruction is independent, a group of sinograms can be reconstructed to image slices in parallel by using the multi-FPGA system [15,16,17]. The parallel processing used to reconstruct the image slices is shown in Figure 2.

In the reconstruction of each single slice of an image, the BPML image reconstruction algorithm is designed to function as several parallel module circuits. This study proposes the use of BPML in an image reconstruction algorithms, designed as parallel module circuits and executed in each FPGA. BPML combines the traditional BPL imaging algorithm with an iterative MEML algorithm, and it shares FPGA characteristic low resource consumption yet has strong parallel processing capabilities [18]. Initially, an image intended for MEML is obtained using the FBP fast imaging algorithm and then inputted into the iterative MEML algorithm [19,20]. BPML can reduce the number of iterations and increase the imaging quality as compared with MEML [21]. Furthermore, several FPGAs can work in parallel to reconstruct several image slices at the same time, from which a 3D image can be built.

### 2.1. Image Reconstruction Algorithms

#### 2.1.1. Obtaining the Initial Image by Using FBP

**Figure 3:**
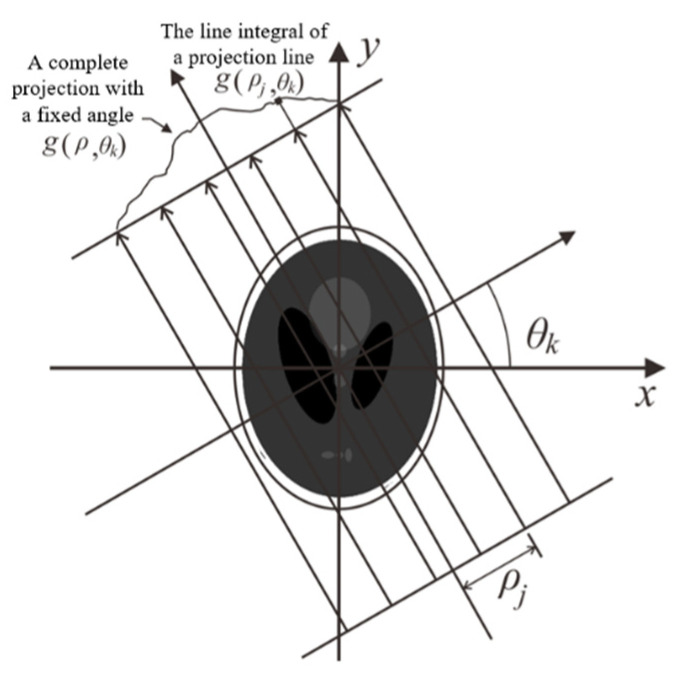
Geometric description of a group of parallel projections.

FBP algorithms build images slices by abstracting sinogram images. Sinograms are used to save image data with *ρ* and *θ* as the rectangular coordinates from a Radon transform, where *θ* is the angle and *ρ* is the distance from the origin to the straight beam. A sinogram is defined as a set of 2D data that can classify all coincident events by (*θ*, *ρ*) [22]. The principle of FBP is demonstrated in Figure 3.

In a rectangular coordinate system, a straight line can be represented by its normal form:

(1) xcosθ+ysinθ=ρ

Parallel straight beams of different angles are projected onto an object and described as *g*(*θ*,*ρ*) (Figure 3). As a function, the Radon transform from the object image to the sonogram data *g*(*θ*,*ρ*) is given by:

(2) $$g(\theta ,\rho )=\int_{-\propto }^{\propto }\int_{-\propto }^{\propto }f(x,y)\delta (x\sin\theta +y\cos\theta -\rho )dxdy$$

According to the characteristics of the impulse function *δ*, the integral is only calculated along the line *x*cos*θ* + *y*sin*θ* − *ρ*.

FBP algorithms are based on the Fourier slice theorem. These algorithms can overcome the image blur problem caused by uniform smearing in the back-projection process. In dependence of *θ*, the Fourier transform of g(θ,ρ) is expressed as Equation (3).

(3) $$G(\theta ,\omega )=\int_{-\infty }^{\infty }g(\rho ,\theta )e^{-2\pi \omega \rho j}d\rho$$

(θ,ω) is a variable in the frequency domain (ρ,θ). Substituting Equation (2) into Equation (3) gives:

(4) $$\begin{array}{l} G(\theta ,\omega )=\int_{-\infty }^{\infty }\int_{-\infty }^{\infty }\int_{-\infty }^{\infty }f(x,y)\delta (x\cos\theta +y\sin\theta -\rho )e^{-2\pi \omega \rho j}dxdyd\rho \\ \;\;\;\;\;=\int_{-\infty }^{\infty }\int_{-\infty }^{\infty }f(x,y)\left[\int_{-\infty }^{\infty }\delta (x\cos\theta +y\sin\theta -\rho )e^{-2\pi \omega \rho j}d\rho \right]\;dxdy\\ \;\;\;\;\;=\int_{-\infty }^{\infty }\int_{-\infty }^{\infty }f(x,y)e^{-2\pi \omega j(x\cos\theta +y\sin\theta )}dxdy \end{array}$$

Substituting *u* = *ω*cos*θ* and *v* = *ω*sin*θ* into Equation (4) gives:

(5) $$G(\theta ,\omega )=\left[\int_{-\infty }^{\infty }\int_{-\infty }^{\infty }f(x,y)e^{-2\pi j(ux+vy)}dxdy\right]$$

By using a 2D Fourier transform, Equation (5) can be transformed into Equation (6) as follows:

(6) G(θ,ω)=F(u,v)=F(ωcosθ,ωsinθ)

where *F*(*u*,*v*) is the 2D Fourier transform of *f*(*x*,*y*).

According to Equation (6), the result of the Fourier transform obtained from a set of projections with a given angle *θ* can build a image slice in the *θ* direction, which corresponds to the Fourier slice theorem.

In solving the image blur problem caused by direct back projection, the projection data are first filtered before calculation. The following formula can be obtained from the 2D inverse Fourier transform:

(7) $$f(x,y)=\int_{-\infty }^{\infty }\int_{-\infty }^{\infty }F(u,v)e^{2\pi j(ux+vy)}dudv$$

Then, by substituting *u* = ωcosθ and *v* = ωsinθ into Equation (7), *dudv* = ωdωdθ can be obtained from the Jacobian matrix. Equation (7) can be transformed into a polar coordinate form as follows:

(8) $$f(x,y)=\int_{0}^{2\pi }\int_{0}^{\infty }F(\omega \cos\theta ,\omega \sin\theta )e^{2\pi \omega j(x\cos\theta +y\sin\theta )}\omega d\omega d\theta$$

From the Fourier slice theorem depicted in Equation (6), the following formula can be obtained:

(9) $$f(x,y)=\int_{0}^{2\pi }\int_{0}^{\infty }G(\theta ,\omega )e^{2\pi \omega j(x\cos\theta +y\sin\theta )}\omega d\omega d\theta$$

According to the central conjugate symmetry characteristics of the Fourier transform, *G*(*θ*,*ω*) is equal to *G*(*θ* + π,−*ω*). Then, Equation (9) can be expressed as:

(10) $$F[f(x,y)]=\int_{0}^{\pi }\int_{-\infty }^{\infty }G(\theta ,\omega )e^{2\pi \omega j(x\cos\theta +y\sin\theta )}\left|\omega \right|d\omega d\theta$$

where |*ω*| is a filtering operator, defined as an oblique filter. As the amplitudes in both directions in Equation (10) are extended to infinity, |*ω*| is not integrable. In solving this problem, the filter operator should be windowed during filtering to ensure that it is only valid within the defined frequency range.

Although the FBP algorithm cannot directly obtain a high-quality PET reconstructed image, FBP is a preferred method for obtaining an initial estimate for MLEM algorithms [23]. Due to the rapidity of FBP algorithms, the basic image slice can be reconstructed in a short time, and the quality of the image reconstructed by FBP will be better than that of the first-iteration images of traditional MLEM, meeting the input conditions as an a-priori estimate. Therefore, the results of FBP reconstruction are input into the fast MLEM algorithm as initial estimates to establish a foundation for iterative estimates of the target images. The image convergence can be sped up by reducing the number of iterations.

#### 2.1.2. Optimizing Image Reconstruction Algorithms of BPML

The initial image pixels are converted into 1D data form, in which *x_n_* is the nuclide concentration in pixel *n*, and *Y_m_* is the count value of coincidence events received by the *m*-th group of detectors. The coincidence events generated by the positrons annihilated at *x_n_* are received by the corresponding detector pairs of *Y_m_*. The number of received coincidence events obeys Poisson distribution by using the parameter of *P_mn_x_n_*, where *P_mn_* is the probability that the coincidence events generated at *x_n_* are counted on the basis of the corresponding detector pairs of *Y_m_*, i.e., the system matrix. The pixels are assumed to be independent of each other and to obey a Poisson distribution.

(11) $$Y_{m}∼Poisson(\sum_{n=1}^{N}p_{mn}x_{n}),n=1,2\cdots N$$

with respect to noise, Equation (11) can be written as:

(12) $$Y_{m}=\sum_{n=1}^{N}p_{mn}x_{n}+e_{m},m=1,2\cdots M$$

where *e_i_* is the noise. According to the Bayesian theorem, the conditional probability of the count value of a coincidence event received under different pixel concentrations can be expressed as a likelihood function.

(13) $$P(Y|x)=\prod_{m}^{M}\frac{{\left(\sum_{n=1}^{N}p_{mn}x_{n}\right)}^{y_{m}}}{Y_{m}!}e^{-\sum_{n=1}^{N}p_{mn}x_{n}}$$

From the perspective of optimization, the maximum likelihood function is equivalent to the log likelihood function, from which it is more convenient to calculate the maximum value.

(14) $$L(x)=\ln P(Y|x)=\sum_{m=1}^{M}\left(Y_{m}\ln\sum_{n=1}^{N}p_{mn}x_{n}-\sum_{n=1}^{N}p_{mn}x_{n}-\ln\left(Y_{m}!\right)\right)$$

where ln(*Y_i_*!) is a constant term of the reconstructed image. If this element is ignored, then Equation (14) can be transformed into:

(15) $$L(x)=\ln P(Y|x)=\sum_{m=1}^{M}\left(Y_{m}\ln\sum_{n=1}^{N}p_{mn}x_{n}-\sum_{n=1}^{N}p_{mn}x_{n}\right)$$

Therefore, the positron annihilation imaging problem under the maximum likelihood function criterion can be reduced to the following constrained optimization problem:

(16) $$\underset{x\geq 0}{\max}L(x)$$

The objective function of the BPML algorithm is written as:

(17) $$\bar{x}=\arg\underset{x\geq 0}{\max}L(x)$$

where $\bar{x}$ is the reconstructed image. Let the function *L*(*x*) be the maximum value of *X*, which is the final result of the reconstructed image. The BPML iterative formula can be obtained by applying the expected maximum algorithm and fixed-point iteration as follows:

(18) $$x_{n}^{k+1}=\frac{x_{n}^{k}}{\sum_{m=1}^{M}p_{mn}}\sum_{m=1}^{M}\frac{Y_{m}p_{mn}}{\sum_{o=1}^{O}p_{mo}x_{o}^{k}}$$

### 2.2. BPML Algorithms Built in FPGA

The mapping of an algorithm involves implementing image-reconstruction algorithms in FPGA. The mapping needs to be transformed into an FPGA systematic structure. Both a pipeline structure and a parallel array exist in FPGA systematic structures. Therefore, whether an algorithm can be reconstructed in FPGA depends on mapping this algorithm into an FPGA systematic structure [24,25].

The pipeline structure originally belongs to the assembly of industrial production lines and serves to decompose a complete assembly process into relatively independent subtasks that can be executed sequentially. The output of the previous step is the input of the next step—it is a one-way process with no feedback or iteration. In FPGA, the BPML imaging algorithm is decomposed into several steps. The reconstruction of a single pixel of an image is fulfilled by decomposing it into step 1 > step 2 > step 3... > step n.

**Figure 4:**
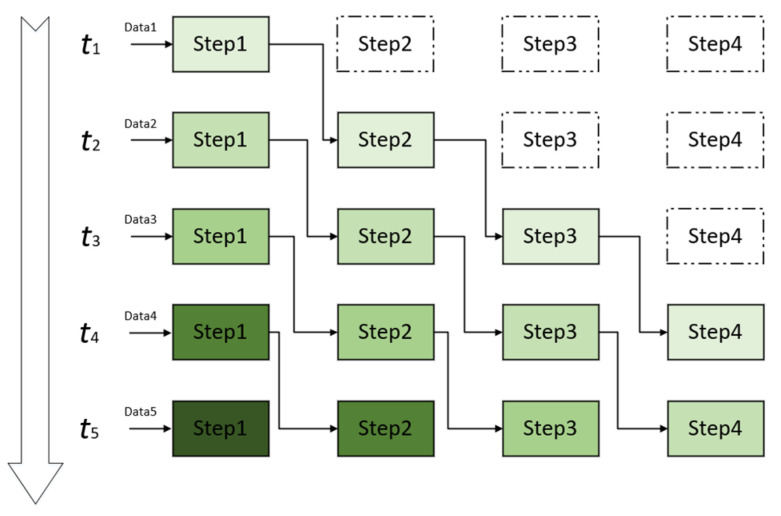
Pipeline design timing diagram.

A pipeline structure is characterized by a continuous input data stream in each step, and the data in the data stream are sequentially inputted into the pipeline as a time sequence (Figure 4). At *t*_1_, we input data 1 to the data streaming to module 1, and then we input it to module 2 at *t*_2_; simultaneously, we input data 2 in the data stream to module 1. With this method, all modules are working at *t*_4_., when pipeline is working at maximum efficiency.

**Figure 5:**
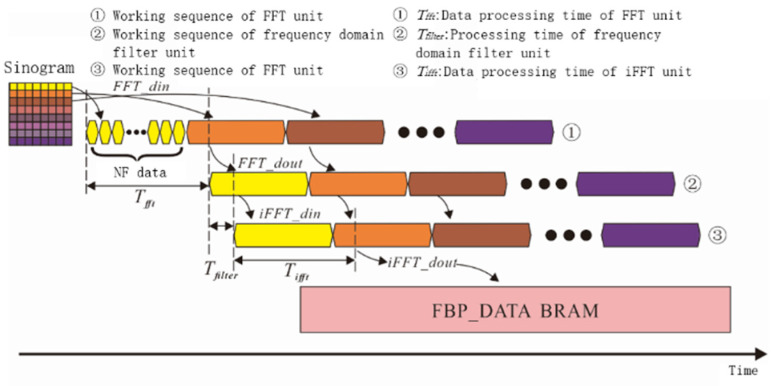
Pipeline sequence diagram of the filter module.

In Section 2.1.2, the BPML algorithm is provided. It uses an a-priori estimate value to enhance the correlation between the iterative image and the target image, which reduces the number of iterations required to obtain a satisfactory image. In this section, FPGA is selected as a hardware accelerator. The BPML algorithm needs, then, to be modified to adapt to the structural characteristics of the FPGA. The initial value of the BPML algorithm is the result of the FBP image reconstruction. The filtering part of the FBP image reconstruction algorithm is divided into sub-modules to ensure parallel operation. The three sub-modules are an FFT unit, a frequency domain filter unit, and an *i*FFT unit. The FFT unit executes FFT(fast Fourier transform) algorithm. These three modules are run in parallel (Figure 5) and are executed at different parts of the pipeline.

**Figure 6:**
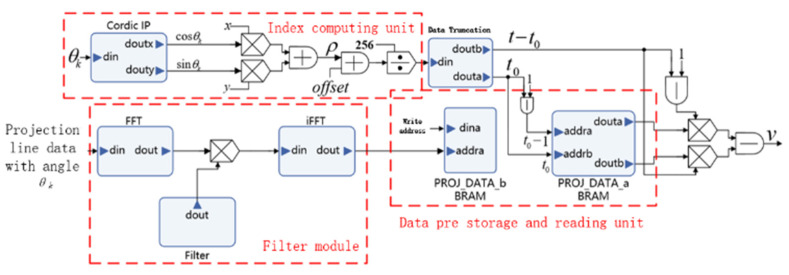
Pipeline sequence diagram of the filter module.

The sinogram data are separated into groups by row. Data in the same row have the same projection angle. Assuming that the *k*-th row data are the projection line data under angle *θ_k_*. Each group of data can be processed in parallel, and the three module circuits can process the different groups of data in parallel. After the first group of data completes the processing in the FFT_din and FFT_dout modules, the results are inputted to the *i*FFT_dout module. Simultaneously, after the second group of data complete the processing in the FFT_din module, the results are inputted to the FFT_dout module. The third group of data are inputted to the FFT_din module. In this method, these three filtering modules can be running in parallel. The parallel array structure of the FBP sub-circuits is shown in Figure 6.

**Figure 7:**
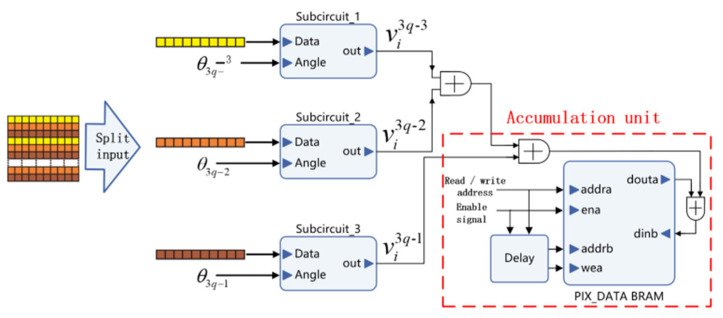
Parallel implementation structural diagram of the prior estimation circuit.

Figure 6 shows the data processing circuits of the projection line data in angle *θ_k_*, representing a row of sinogram data. The data are initially filtered in the frequency domain by the filter module and then buffered in PROJ_DATA_b BRAM (block random access memory), which is writable. Simultaneously, the index calculation unit calculates and obtains the storage address of the data from angle *θ_k−_*_1_. Furthermore, the data from *θ_k−_*_1_ are input into PROJ_DATA_a BRAM, which executes a reading function. After the projection data under a certain angle are processed, the two sets of BRAM exchange their reading or writing functions and iterates to the next angle projection data without any delay. The division by sub-circuit ensures the continuity of the circuit’s sequence with high efficiency. A parallel array structure is designed by copying several sub-circuits (Figure 7).

The parallel array is composed of three groups of the same sub-circuits. The sinogram parameters are divided into groups according to the row vectors. The data of *q*-th group include parameters in three angular types, namely, *3q-1*, *3q-2*, and *3q-3*. The angle data and its corresponding projection data are inputted into the sub-circuit. The output of *v_i_^3q-3^* is the *i*-th reconstruction pixel in the image obtained from the projection data of *θ_3(q-1)_*. Then, the reconstruction values of the same pixel from all of the data with different projection angles of *θ* are added, and a pixel of the reconstructed image is finally obtained.

## 3. Implementation of Edge Detection in FPGA

The image reconstructed by the BPML algorithm is a tomographic image. The internal image of the industrial part can be displayed. If defects exist on the inner surface of the cavity, it may be visible at the edge of the object in the image [26]. The edge of the inner area in a tomographic image is assessed to judge defects. The Sobel operator, which is usually used in edge detection, can only detect edge information in the 0° horizontal direction and 90° vertical direction, suggesting a potential loss of edge information in the other directions [27,28]. In this study, the multi-directional improved Sobel operator, instead of the traditional two-directional Sobel, operator is used.

FPGA requires 2D matrix simulation when processing spatial images. This is implemented via data sequence alignment. The image data are input into FPGA in the form of a data stream. While three adjacent rows of data are obtained simultaneously, the data need to be registered and then output after a specified delay. For a 3 × 3 mask structure, the input data stream is generally combined with two cascaded shift registers to complete the timing alignment of the three rows of data. The shift register is provided by the Shift_RAM IP core. The IP core can shift a specified number of bits within one clock cycle and output the registered data based on the first-in first-out rule.

**Figure 8:**
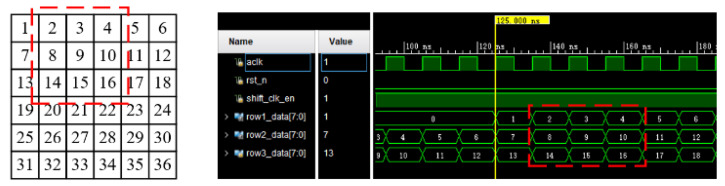
Parallel implementation structural diagram of the prior estimation circuit.

Figure 8 shows an example of 6 × 6 data-stream image. The correctness of the timing alignment is verified using a Vivado Simulation.

**Figure 9:**
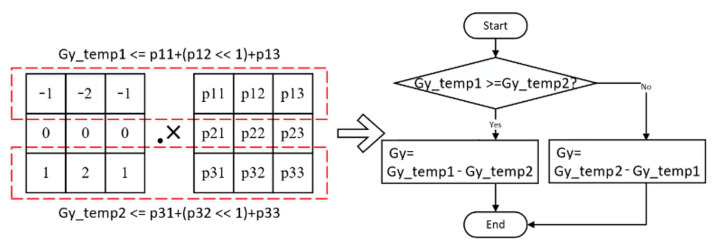
Diagram of the improved Sobel vertical gradient calculation method.

The above-mentioned timing alignment method is also applicable in selecting the median filter input data. As the mask weighting coefficients of the four-directional Sobel operator are all composed of one, two, and zero, multiplying the coefficient by two in the direction gradient calculation of the FPGA can be realized by shifting to the left, thus avoiding the use of multipliers. Take the Sobel mask as an example, its logical discrimination relationship is shown in Figure 9.

**Figure 10:**
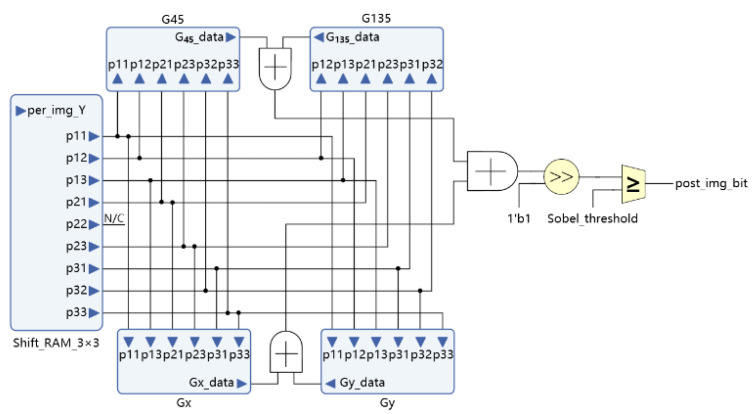
Diagram of the improved Sobel vertical gradient calculation method.

The four-directional gradient calculation is completed in parallel. The weighting coefficient of each direction mask determines the input of each gradient direction’s calculation unit. Figure 10 illustrates the parallel circuits’ structural diagram of the four-directional Sobel operator.

**Figure 11:**
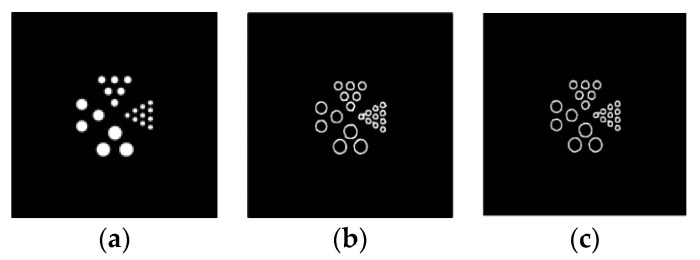
Detection results based on two-directional detection and four-directional edge detection. (**a**) Four sectors of a Derenzo image. (**b**) Result of two-direction Sobel algorthim (connectivity ratio 0.6355). (**c**) Result of four-direction Sobel algorthim (connectivity ratio 0.4844).

A four-sector Derenzo image is used to verify the method. The image connectivity ratio is used to compare the results of the traditional Sobel operator and the improved four-directional Sobel operator in terms of edge detection, which is shown in Figure 11. The connectivity ratio of the four-directional Sobel operator is 0.484, which is better than the result from the two-directional Sobel operator.

## 4. Experiments

### 4.1. Preparation for Experiments

**Figure 12:**
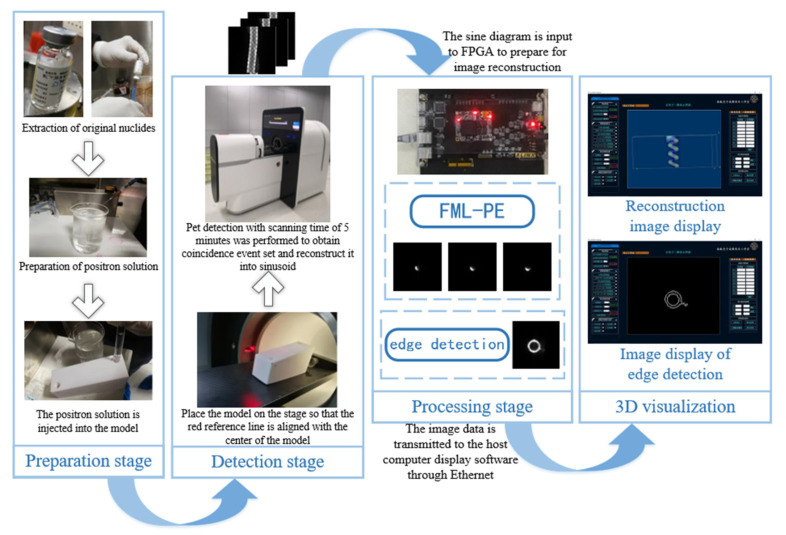
PET experimental process.

Figure 12 illustrates the three stages (preparation, detection, and processing) in the experiments.

The preparation stage includes preparing a positron solution and injecting the positron solution to the experimental model. The positron solution is a mixture of 18F-labeled original nuclides with water at a certain proportion. In consideration of the nuclides’ radioactivity, the positron solution used in the experiments was minimized to that necessary to meet testing requirements.

Coincidence events were obtained from the PET scanning of the object, and the testing data were transformed into a sinogram. A sinogram is a type of 2D matrix saving the data of coincidence events with different distances in varying directions. The sinogram is input into the FPGA, and the image slices are reconstructed using the BPML algorithms. Additionally, objects’ edges in the image are detected in the FPGA to help detecting the defects.

The PET detector used in the experiments was a Trans-PET Explorist-180. Its basic unit block is a 13 × 13 crystal strip array. Four block units form a set of heads in the axial direction, and 24 sets of heads arrayed as a ring form one of the PET’s detecting ring. In this study, the GATE (Geant4 Application for Tomographic Emission) simulation platform was used. The parameters of the GATE simulation detectors represent the real PET detectors. The parameters used in the PET and GATE simulations are the same, and are shown in Table 1. The resolution in all three directions is 0.5 mm in good conditions.

### 4.2. A Experiment for a Model

#### 4.2.1. Completing the Experiment

**Figure 13:**
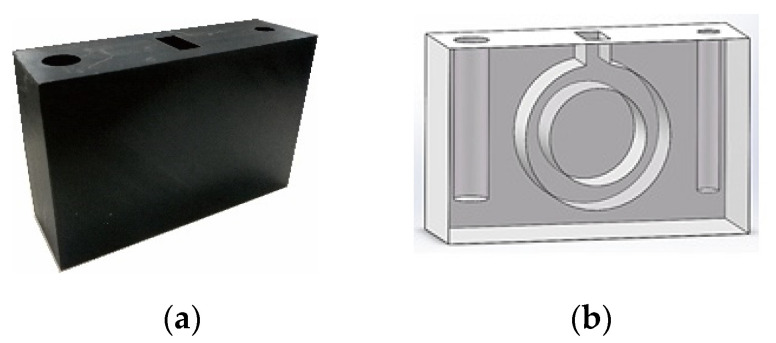
The experimental model. (**a**)Physical structure of model 1. (**b**) Solid work design of model 1.

In this experiment, the testing object is a straight channel and circular pipe model, which is shown in Figure 13. The diameters of the thick and thin straight channels are respectively 15 and 9 mm. The annular pipe designed in this model can help to verify the image reconstruction algorithm for curved structures. The inner diameter of the annular pipe is 20 mm, and the outer diameter is 30 mm. The concentration of the positron solution used in the experiment is 325 kbq/cc, and the scanning time of PET in this experiment is 5 min.

**Figure 14:**
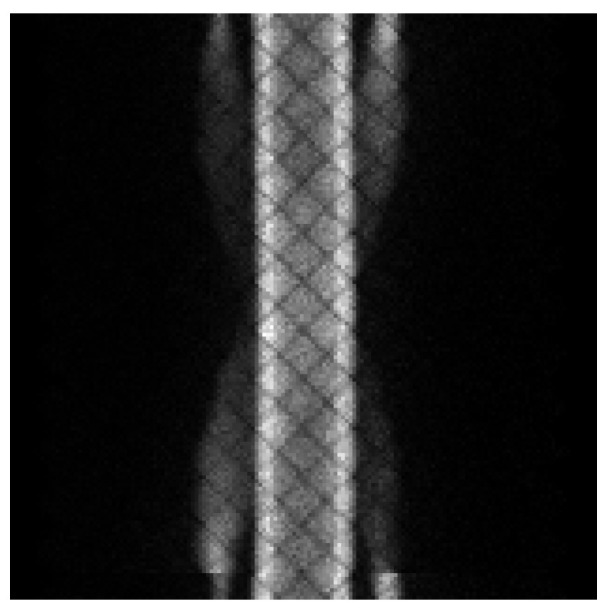
The 25th image slice.

The coincidence events detected by PET are processed and saved in sinograms. Since the edges of the test object do not contain areas of interest, we randomly selected slice 25 as a representation from those sinograms containing material contrasts (Figure 14).

**Figure 15:**
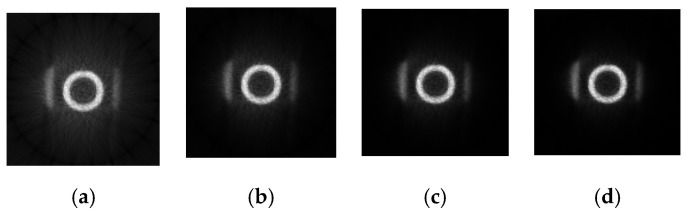
The reconstructed images by BPML algorithm with one to four iterations in FPGA. (**a**) One iteration. (**b**)Two iterations. (**c**) Three iterations. (**d**) Four iterations.

Figure 15 shows the reconstructed images (size: 128 × 128) by BPML on FPGA.

**Figure 16:**
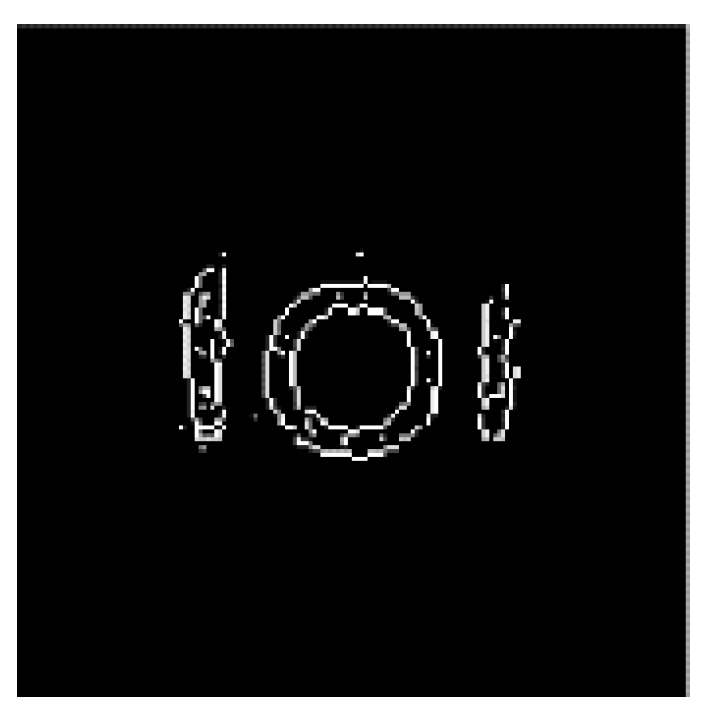
Edge detection result.

After four iterations, the reconstructed images are processed by the improved four-directional edge detection. The results are shown in Figure 16, which verify the effectiveness of the image edge detection.

**Figure 17:**
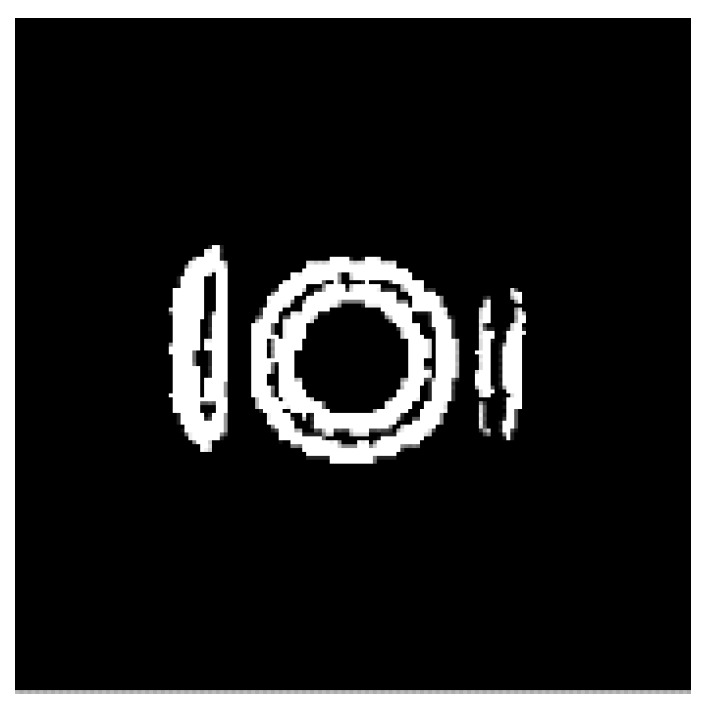
Edge refinement.

The edge detection completes the extraction of the main boundary information of the image, and it also removes the ringing fringes around the image. However, the detected edges are extremely thick, due to the deficiency of the Sobel operator itself and the limited resolution of the reconstructed image (128 × 128). Therefore, edge refinement is used to obtain the edge information more accurately. The refined image is shown in Figure 17.

The image processed after edge refinement can be used to depict the outline of the object. Figure 17 clearly shows the internal structure of the model. Although the image, after edge refinement, shows a few bright spots inside the measured object, the overall image quality is improved.

#### 4.2.2. Algorithm Execution Time Analysis

**Figure 18:**
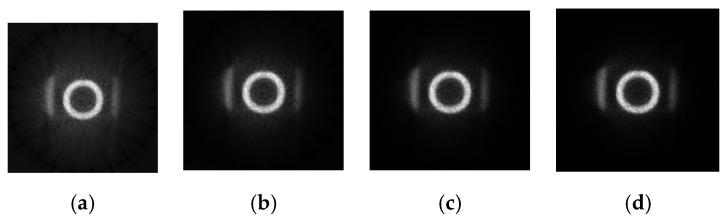
The reconstructed images by the BPML algorithm with one to four iterations in the CPU. (**a**) One iteration. (**b**) Two iterations. (**c**) Three iterations. (**d**) Four iterations.

The same image reconstruction and processing algorithms are run in the CPU, and the reconstructed image is shown in Figure 18.

**Figure 19:**
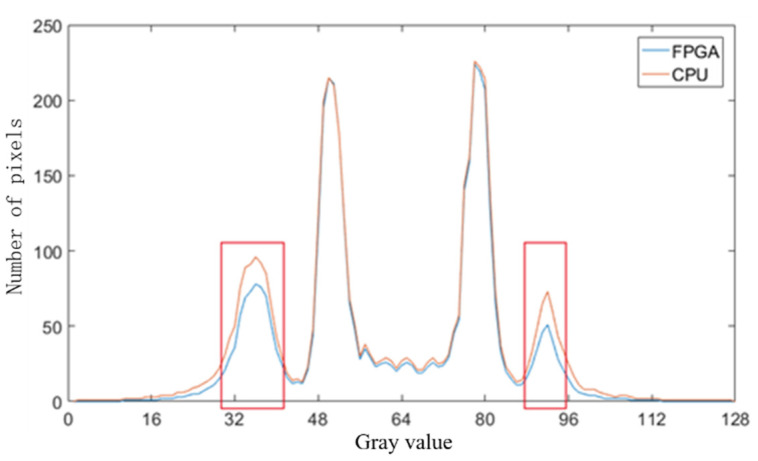
Comparison of the quality of images reconstructed in FPGA and CPU.

Comparing Figure 18 with Figure 15 indicates that the whole quality of image slices reconstructed by FPGA is close to that reconstructed by CPU. To show the details of the two images, the fourth iteration images reconstructed by the FPGA and CPU are compared. The pixels values in the 64th row of the images are counted in Figure 19. The quality of the FPGA images is similar to that of the CPU images, except in the strong edge (the red square in Figure 19). The values of FPGA images are lower than those of CPU images within the red square because of precision truncation, which can suppress the BPML ringing effect.

**Figure 20:**
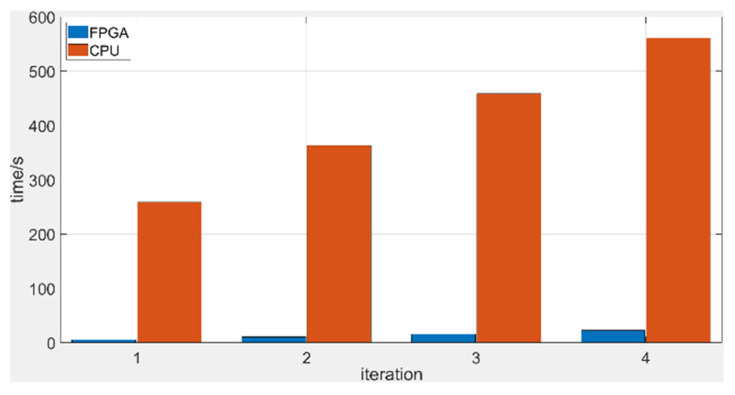
Comparison of the images’ reconstruction times in FPGA and CPU.

The average execution times for image reconstruction by the FPGA and CPU are compared in Table 2 and Figure 20. The execution time for BPML on the FPGA for a single image was 0.59 s. Based on the workload in the parallel strategy, only 22.95 s is required to reconstruct 104 image slices. Table 2 and Figure 20 show that image reconstruction by the FPGA is 45 times faster than that by CPU.

#### 4.2.3. Internal Imaging Experiment of Hydraulic Parts

**Figure 21:**
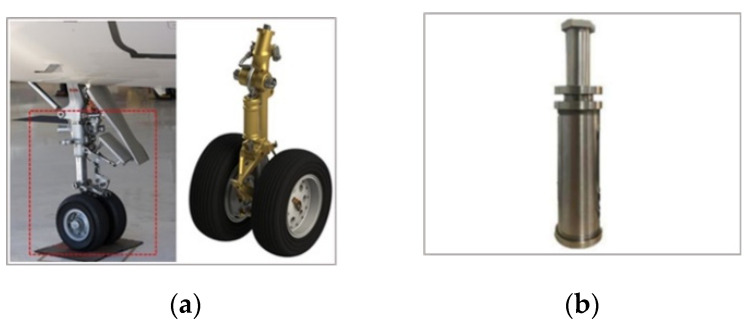
Hydraulic parts. (**a**) Application of hydraulic parts in aircraft landing gear. (**b**) The tested hydraulic part.

**Figure 22:**
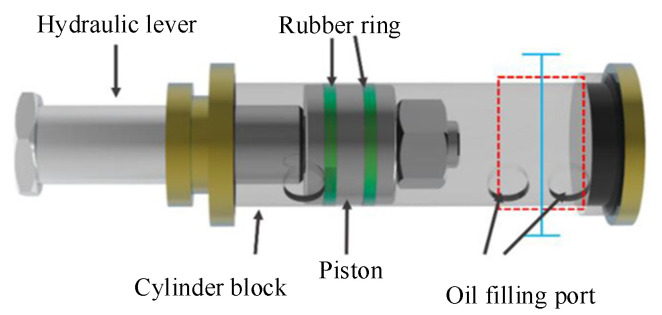
The structure of the tested hydraulic part.

Hydraulic technology is based on the Pascal principle and is one of the key technologies in modern transmission and control. It has the advantages of a large power-to-weight ratio, small volume, little inertial motion and a fast reaction speed. Therefore, hydraulic parts are widely used in many fields. Figure 21a shows the application of hydraulic parts in aircraft landing gear. As a result of the high oil pressure in the inner cavity of a hydraulic component, the inner defects may appear in, which will affect performance. In this study, the second experiment tests the inner cavities of hydraulic parts. Figure 21b shows the actual hydraulic part used in this experiment. The structure of the hydraulic part is shown in Figure 22. The wall thickness of the hydraulic part is 5 mm, the outer diameter is 73 mm and the material is made of stainless steel. In order to verify the effectiveness of the proposed scheme in detecting internal defects, a metal wire, of 2-mm sectional diameter, was placed in the red frame area in Figure 22, and then the hydraulic part was tested. The experiment was performed according to the procedure shown in Figure 12. The experimental process was as follows: (1) place a section of metal wire with a diameter of 2 mm into the hydraulic part; (2) fully mix 18F labeled radionuclides with hydraulic oil and mix the solution evenly with a mixer; (2) inject 310 ml of labeled hydraulic oil inside the hydraulic component through the hydraulic opening; the activity of the solution should be 2.4 mCi; (3) fix the hydraulic part in the center of the PET scanning area, and set the detection time to 10 min.

**Figure 23:**
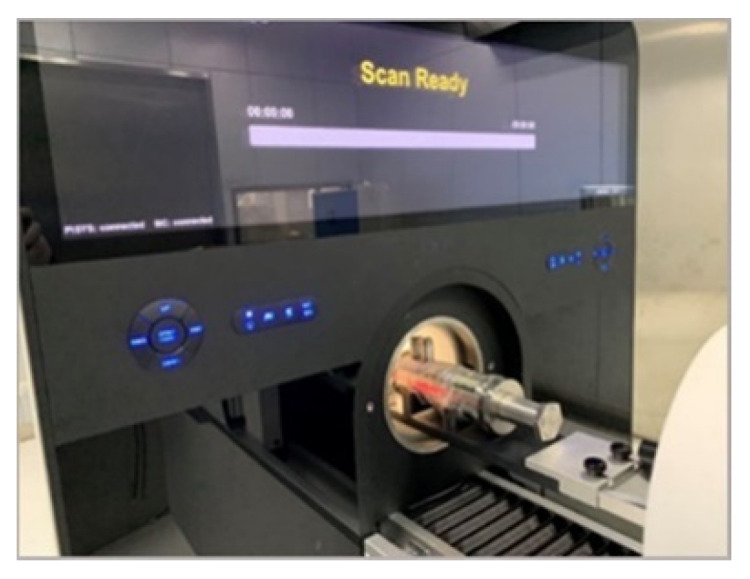
Scanning the hydraulic part.

**Figure 24:**
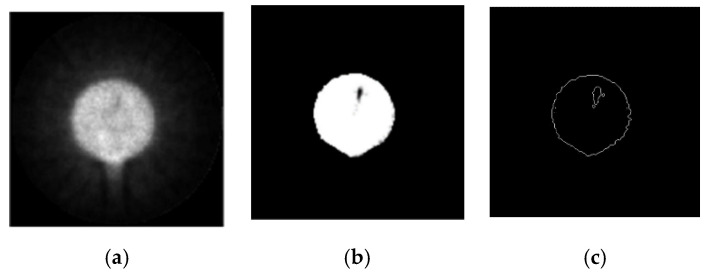
The 150th reconstructed image slice. (**a**) The original reconstructed image. (**b**) The 150th image after processing. (**c**) The 150th image after edge extraction.

The hydraulic part was scanned in PET, which is shown in Figure 23. After scanning and image reconstruction, a group of image slices are built. The 150th image slice was used as an example and is shown in Figure 24a. γ photons are scattered more when passing through stainless steel than through nonmetallic materials. However, after filtering, the reconstructed image was satisfactory. It is approximately at the position of the blue line in Figure 22.

**Figure 25:**
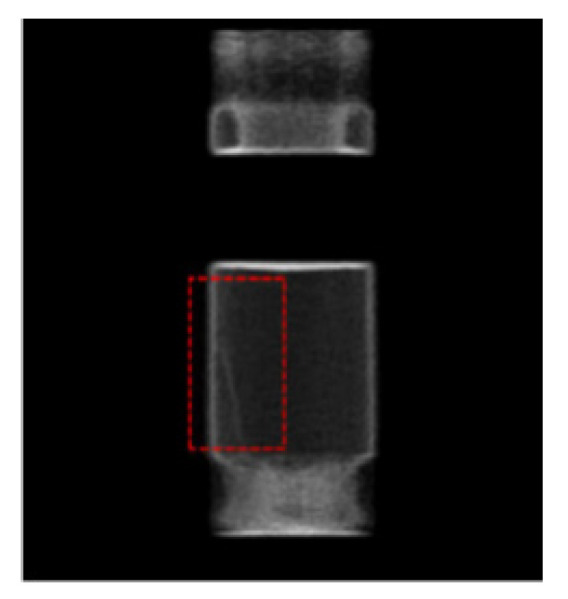
The reconstructed 3D testing image of the hydraulic part.

After image processing, the defect in the 150th image slice is clearer, as shown in Figure 24b. The result, after edge extraction, is shown in Figure 24c. A set of image slices were built into a 3D image, which is shown in Figure 25. As visible in the 3D image, the metal wire is clearly shown in the internal image of the hydraulic part.

## 5. Conclusions

Based on a new method of γ photon imaging to detect the internal defects of complex industrial parts, a parallel working scheme was designed and implemented in FPGA. The speed of positron image reconstruction was accelerated, attributable to two aspects: optimization of the reconstruction algorithm and the use of an FPGA parallel implementation. Aiming to address the shortcomings of iterative reconstruction algorithms, such as a long execution time while iterating, a fast MLEM optimization algorithm (BPML) was proposed. BPML introduces an a-priori estimate value to enhance the correlation between the iterative image and the target image, which reduces the number of iterations required to obtain a satisfactory image. FPGA was selected as a hardware accelerator, and each functional unit of the algorithm was constructed by modular design in Vivado. The IP core is flexibly called to implement the complex functions. The subcircuit of the image reconstruction algorithm was designed with a pipeline structure, and many identical subcircuits constituted a parallel, accelerated circuit. According to the characteristics of the reconstructed image, a multi-directional Sobel edge detection optimization algorithm, based on a fast median filter, was implemented by FPGA to rapidly extract edge information and complete the preprocessing operations of the image reconstruction. The results of our experiments show that both the improved image reconstruction algorithm and improved edge detection algorithm had better performance compared with the respective classic algorithms. Two experiments were designed and conducted. The experiment results verified that the proposed method could detect 2 mm×2 mm defects inside industrial parts. By transplanting the algorithm to FPGA, the detection speed was increased by more than 20 times while maintaining the same detection accuracy. Furthermore, the structures of FPGAs are flexible, and the number of deployed FPGA chips is scalable, which provides room tofurther improve the parallel detection speed.

## Figures and Tables

**Table 1:** Detector Parameters For A Single Ring.

Parameters	Values
detector inner diameter	190 mm
axial length	108 mm
spatial resolution	0.99 mm
Energy resolution	12.83% at 511 KeV
Time resolution	1.53 ns
sensitivity	7.12% at 350–650 KeV

**Table 2:** Comparison of consumption time of BPML in FPGA and CPU.

Platform (Type, Frequency)	Consumption Time for Reconstructing 52 Slices			
	One Iteration	Two Iterations	Three Iterations	Four Iterations
FPGA (XC7A100T, 125 MHz)	5.37	10.79	16.12	22.95
CPU (Core i7-4790, 3.6 GHz)	259.38	362.73	458.75	561.54
Acceleration ratio	48.3×	33.6×	28.5×	24.4×
